# A Cost-effectiveness Analysis of Brentuximab Vedotin in Relapsed or Refractory Systemic Anaplastic Large Cell Lymphoma

**DOI:** 10.36469/9820

**Published:** 2016-10-26

**Authors:** Margaret Hux, Denise Zou, Esprit Ma, Peter Sajosi, Andreas Engstrom, Ross Selby, Eugene Benson, Andrew Briggs, Vijayveer Bonthapally

**Affiliations:** 1 ICON plc, Vancouver, Canada; 2 Center for the Evaluation of Value and Risk in Health, Institute for Clinical Research and Health Policy Studies, Tufts Medical Center, Boston, MA, USA; 3 Global Pricing, Market Access and Health Economics Millennium Pharmaceuticals Inc., Cambridge, MA, USA, a wholly owned subsidiary of Takeda Pharmaceutical Company Limited; 4 Market Access and Health Economics, Takeda Pharma AB, Stockholm, Sweden; 5 Market Access, Takeda UK Ltd., Bucks, United Kingdom; 6 ICON plc, New York, NY, USA; Institute of Health and Wellbeing, University of Glasgow, Glasgow, UK

**Keywords:** incremental cost-effectiveness ratio, quality-adjusted life year, chemotherapy, systemic anaplastic large cell lymphoma, brentuximab vedotin, cost-effectiveness

## Abstract

**Objective:** To evaluate the cost-effectiveness of brentuximab vedotin in patients with R/R sALCL from a UK NHS perspective.

**Methods:** A partitioned survival model used clinical outcomes for brentuximab vedotin from the pivotal phase-2 single-arm trial of brentuximab vedotin in 58 patients with R/R sALCL (SG035-0004; NCT00866047), over a lifetime (30-year) time horizon. Comparison with conventional chemotherapy was based on data from the Canadian British Columbia Cancer Agency registry from 40 patients starting salvage chemotherapy after front-line treatment between 1980 and 2012. Survival was extrapolated using parametric distributions, with brentuximab vedotin risk after the trial period assumed equal to conventional chemotherapy. Other modelling assumptions were based on a systematic literature review and clinical expert opinion.

**Results:** Based on statistical extrapolation, brentuximab vedotin was associated with 3.1 years longer duration in the progression-free survival health state and an overall survival improvement of 5.4 years, prior to discounting. In addition, brentuximab vedotin was associated with 2.5 quality-adjusted life years (QALYs) gained at a total incremental cost of £88 556, resulting in an incremental cost-effectiveness ratio (ICER) of approximately £35 400. Sensitivity analyses of alternative model assumptions provided ICERs ranging from approximately £28 100 to £61 900. Comparing only first-line salvage patients reduced the ICER to £26 800 per QALY gained. Conversely, considering only patients with Eastern Corporative Oncology Group performance status of 0 or 1 increased the ICER to approximately £38 200. At a willingness-to-pay threshold of £50 000, the estimated probability that brentuximab vedotin is cost-effective compared with conventional chemotherapy was 86.5%.

**Conclusion:** Compared to conventional chemotherapy, and considering the full survival period, brentuximab vedotin may provide a valuable treatment choice for patients with R/R sALCL, a population with limited therapeutic options.

## BACKGROUND

Systemic anaplastic large cell lymphoma (sALCL) is an aggressive T-cell non-Hodgkin lymphoma (NHL) that primarily involves sinus areas of the lymph nodes.[1,2] As a subgroup of peripheral T-cell lymphoma (PTCL), sALCL is a rare disease, representing approximately 2–3% of NHL cases in adults and 10–15% in children.[1] The incidence of ALCL is estimated to be <1 case per 100 000 persons[3], which is below the level specified by the European Medicines Agency (EMA; 5 cases in 10 000 persons) for designation as an orphan disease.[4]

First-line therapy for adult patients with sALCL is often a multi-agent, anthracycline-containing regimen, most commonly CHOP (cyclophosphamide, doxorubicin, vincristine, and prednisone), and concurrent radiation.[5] This treatment regimen has been shown to produce objective responses in 86% of patients with anaplastic lymphoma kinase (ALK)-positive sALCL which is associated more favorable survival[2] and in 68% of patients with ALK-negative disease. Overall survival (OS) rates at 8 years are 82% and 49%, respectively.[6]

However, around 70% of patients are refractory to or relapse following front-line treatment.[5] Currently, there is no consensus on recommendations for treatment in these patients and their long-term prognosis remains poor. Although it is difficult to estimate due to the rarity of sALCL, 5-year OS is estimated to be approximately 34% in adults with PTCL (including ALCL), and 57–65% in children and adolescents with ALCL[7-9], with an estimated median OS of 5.5 months following relapse or progression after primary therapy.[10] Thus, there is a clear unmet need to develop new treatments for relapsed or refractory (R/R) sALCL and to evaluate their costs and effectiveness to support patient access.

Salvage therapy for patients with R/R sALCL can vary considerably. Treatment guidelines support use of platinum-based chemotherapy at relapse, with stem cell transplantation (SCT) in patients with chemotherapy-sensitive disease, and gemcitabine and platinum used in second and third line.[5] Conventional platinum-based regimens such as ICE (ifosfamide, carboplatin, and etoposide), ESHAP (etoposide, methylprednisolone, cytarabine, and cisplatin) and Gem-P (gemcitabine, cisplatin, and methylprednisolone) are recommended in treatment guidelines and are widely used.[5,11] However, these guidelines also refer to the lack of robust evidence informing treatment choice, the low complete response rates and short duration of response observed with chemotherapies, and the dismal prognosis of relapsed patients.[5,11]

Brentuximab vedotin (ADCETRIS®, Seattle Genetics, Inc., Bothwell, WA, USA) is an antibody–drug conjugate with CD30-specific anticancer activity. In a pivotal phase-2, open-label, single-arm, multicenter study (SG035-0004; NCT00866047), 58 patients with R/R sALCL and good performance status, median age 52 years, were given brentuximab vedotin 1.8 mg/m2 by intravenous infusion every 3 weeks. Objective responses were achieved in 86% of patients (59% complete remission [CR] and 27% partial remission [PR]).[12] Grade 3 or 4 adverse events (AEs) observed in ≥10% of patients were neutropenia (21% of patients), thrombocytopenia (14%), and peripheral sensory neuropathy (12%). Overall, brentuximab vedotin demonstrated significant clinical activity in both ALK-positive and ALK-negative ALCL, and was associated with manageable toxicity.[12-14] Based on these results, brentuximab vedotin was granted accelerated approval by the United States Food and Drug Administration for treatment of patients with sALCL after failure of at least one multi-agent chemotherapy regimen[15], and conditional approval from the EMA for the treatment of adult patients with R/R sALCL.[16]

For patients to be able to access new therapies, data are required that quantify their overall clinical benefits and incremental costs compared with current treatments. As the pivotal phase-2 study of brentuximab vedotin was a single-arm, non-comparative study, direct comparative data are not available.[12-14] A systematic literature review, conducted as part of this study, found that there is a paucity of published studies of chemotherapy treatment in patients with R/R ALCL, which limited the availability of adequate data to support development of the economic evaluation model. The Canadian British Columbia Cancer Agency (BCCA) registry with a cohort of patients with R/R sALCL provided the best available data, which align with data from the brentuximab vedotin trial, to inform long-term survival outcomes in this patient population.

The objective of this study was to develop a cost-effectiveness model comparing brentuximab vedotin with conventional chemotherapies for patients with R/R sALCL, from a UK National Health Service (NHS) perspective. The model is built to reflect practice for these patients in the UK setting, but can easily be adapted to represent other jurisdictions.

## METHODS

### Model Structure

We estimated the cost-effectiveness of brentuximab vedotin in R/R sALCL using an excel-based partitioned survival model (area under the curve) comprising three health states (progression-free survival [PFS], post-progression survival [PPS], and death), over a lifetime (30-year) time horizon. Parametric survival functions were used to estimate the proportion of the cohort remaining free of endpoints that defined the relevant health states. Time in the PFS state was estimated directly from the PFS curves per investigator review. Time in the PPS state was estimated by the difference between the PFS and OS curves at each time point.

Costs and clinical outcome benefits were estimated at fixed time points based on the proportion of the cohort in each of the three health states at a given time. The model utilized a 21-day cycle and was run for a life-time (30-year) time horizon over which costs and health outcomes were evaluated, with a discounting rate of 3.5% per annum.

Based on clinical expert opinion along with findings from a targeted literature review and treatment guidelines, in the base case, conventional chemotherapies, including ICE, ESHAP, and Gem-P, were each assumed to be received by one-third of sALCL patients in the United Kingdom.[5,17,18]

### Data Sources

Individual patient data from SG035-0004 were used to inform the efficacy and safety of brentuximab vedotin. At the time of the analysis, with a median observation time of 33.4 months (range 0.8–45.6) the objective response rate was 86%, with CR achieved in 59% of patients.[12]

The estimated long-term survival outcomes with conventional chemotherapy were based on the BCCA registry, with a sample of 40 patients with sALCL, who started first-line salvage chemotherapy between 1980 and 2012 and were followed for up to 20 years through patient charts. Data of the BCCA registry were provided by Dr Joseph M. Connors, Clinical Director at the BC Cancer Agency Centre for Lymphoid Cancer (data on file).

Resource use and costs associated with brentuximab vedotin and conventional chemotherapies were estimated using UK NHS cost data, based on the 2014 cost year. Health benefits were expressed in terms of life-years (LYs) gained and quality of life-adjusted LYs (QALYs) gained. Cost-effectiveness results were presented as incremental cost per LY gained and incremental cost per QALY gained.

## Indirect Comparison

An indirect comparison between brentuximab vedotin (SG035-0004 data) and conventional chemotherapies (BCCA registry data) was used since direct comparative evidence was not available. This comparison has the potential for bias due to differences in the study design and differences in patient baseline characteristics; therefore, potential confounding variables were identified and addressed through sensitivity analyses. Baseline characteristics are shown in Table 1. Baseline characteristics considered for potential bias were: line of salvage therapy, Eastern Co-operative Oncology Group (ECOG) performance status, prior SCT, B-symptoms, age, and ALK status.

**Table 1.** Patient Demographics, Disease Characteristics and Risk Factors

| Characteristic | Bretuximab vedotin[12] | Conventional chemotherapy |
|---|---|---|
| Number of patients, N | 58 | 40 |
| Median age, years (range) | 52 (14–76) | 56 (16–82) |
| ECOG PS, n (%) | | |
| ≤1* | 57 (98) | 26 (65) |
| >1 | 1 (2)† | 14 (35) |
| ALK status, n (%) | | |
| Negative | 42 (72) | 24 (73)‡ |
| Positive | 16 (28) | 9 (27)‡ |
| First-line salvage (1 prior chemotherapy)*, n (%) | 17 (29) | 40 (100) |
| Prior ASCT*, n (%) | 15 (26) | 0 (0) |
| Baseline B symptoms, n (%) | 17 (29) | 9 (23) |

Source: Data for brentuximab vedotin are taken from SG035-000412; data for conventional chemotherapy are taken from the BCCA registry (data on file).

ALK, anaplastic lymphoma kinase; ASCT, autologous stem cell transplant; ECOG PS, Eastern Co-operative Oncology Group performance status.

*$p<0.001$ between groups, all other categories were p=NS between groups

†Inclusion criteria for SG035-0004 required all patients to have an ECOG PS of 0 or 1 (1 patient with ECOG performance status of 2 was enrolled in violation), while 35% of patients treated in the Canadian registry had an ECOG PS ≥1.

‡7 patients were not tested for ALK status.

Although more patients in SG035-0004 had received prior autologous SCT (26% vs 0% of registry patients), the analysis of SG035-0004 showed that patients with prior SCT had a better survival, which contradicts the generally accepted clinical view. Thus, given the small patient sample size, no statistical adjustment has been made to account for this discrepancy between the two groups. Presence of B-symptoms (29% vs 23% of patients), median age (52 years vs 56 years) and negative ALK status (72% vs 73% of patients) did not differ significantly between the treatment populations and were not considered further for potential bias.

Line of therapy and ECOG performance status were expected to impact survival outcomes and were considered in the analysis. While all patients in the registry were followed from first-line salvage therapy, only 29% of patients in SG035-0004 had no prior salvage therapy. The BCCA patients had a mixture of ECOG performance status from 0 to 4, whereas all but one patient in SG035-0004 had an ECOG performance status 0 or 1. Since the sample size was not sufficient to conduct analyses adjusted for both factors, and the direction of bias due to these two factors is opposite, a naïve comparison was considered to represent the best overall estimate and the effect of this factor was explored using sensitivity analyses on cost-effectiveness.

### Extrapolation of Clinical Outcomes (PFS and OS)

Four parametric models (exponential, Weibull, lognormal, and Gamma) were considered and fitted to the data for both datasets. Based on Akaike Information Criterion and Bayesian Information Criterion statistics, and visual inspection of an overlay of the Kaplan–Meier survival curves and fitted parametric functions, the following distributions were chosen for the base case analysis for both PFS and OS: gamma distribution for all brentuximab vedotin-treated patients, exponential distribution for first-line brentuximab vedotin-treated patients, and lognormal distribution for all chemotherapy-treated patients and chemotherapy-treated patients with good performance status.

Since median age was 52 years and 56 years for the brentuximab vedotin and chemotherapy cohorts respectively, a maximum lifetime horizon of 30 years was used to extrapolate survival outcomes. In order to remain conservative, predicted survival curves based on the fitted distributions for the brentuximab vedotin group were projected assuming the risk of progression or death was equal to that for conventional chemotherapy observed from the BCCA dataset beyond the SG035-0004 maximum follow-up period (approximately 55 months).

### Adverse Events

The model considered the cost and quality-of-life (utility) impact of treatment-related AEs. The frequencies of brentuximab vedotin-related AEs were obtained from SG035-0004. Since the BCCA registry does not have information on AEs, the frequencies of AEs for the comparator chemotherapies were estimated from published clinical trials of these therapies in patients with relapsed aggressive lymphoma or NHL.

Only grade 3–4 AEs that occurred in more than 5% of patients (or cycles) were included. AEs associated with alloSCT were assumed only to occur once per patient, and the cost and utility implications of the associated AEs were assumed to occur on the day of transplant.

### Utilities

Utilities of different ALCL health states were obtained from a previous study that elicited values from 100 members of the UK general public using the Time Trade-Off (TTO) method[19], with disease state descriptions (complete response, partial response, stable disease, and progressive disease) based on a literature review, and patient and clinician interviews.

To capture the impact of the different response rates on quality of life, the utility level of the PFS state was weighted according to the proportion of patients in each response category for brentuximab vedotin and conventional chemotherapy. The response rates of patients receiving brentuximab vedotin were obtained from SG035-0004.[12] As no response rates were available from the BCCA registry dataset, response data reported for 89 patients with PTCL from the same database as the BCCA registry were applied.[10]

Disutility values for AEs were estimated from a targeted literature search, based on the assumption that the utility decrement associated with an AE was not dependent on the cancer type or stage of the population in which the utility was measured.

### Resource Use and Costs

Costs included drug acquisition and administration, co-medications, radiotherapy, treatment monitoring, management of AEs, subsequent SCT costs, and supportive care before progression or during progressive disease. Estimates of resource use were obtained from product monographs, literature[20,21] and clinical expert opinion. Drug costs were taken from the British National Formulary, April 2015[22], and unit costs for all other resources were sourced from the NHS Reference Costs 2013–2014.[23]

Drug wastage was considered for brentuximab vedotin but not for conventional chemotherapies because sALCL is a rare disease and unlikely to have vial sharing for brentuximab vedotin, whereas conventional chemotherapy drugs may be used for other cancers.

Brentuximab vedotin was administered in SG035-0004 as a single infusion on an outpatient basis, once every 21 days for up to 16 cycles.[12] The mean number of cycles received was 8.2 (standard error, 0.63). For conventional chemotherapy regimens, inpatient stay was assumed to be required based on clinical expert opinion and the associated costs were included in the model. Fifteen percent of patients in the conventional chemotherapy group were assumed to require radiotherapy as adjunct to their chemotherapy based on clinical input and the BCCA dataset. This was allocated as a one-off cost at the beginning of treatment.

The total cost of SCT was estimated as a weighted average of the cost of allogeneic procedures and autologous procedures based on the assumption that 50% patients receive alloSCT in the brentuximab vedotin arm (based on SG035-0004 data)[12] and 20% proceed to alloSCT in the conventional chemotherapy arm (based on the BCCA registry data).

A Canadian micro-costing study by Lee et al. in diffused large B-cell lymphoma (DLBCL)[20] was applied to estimate costs of disease management post progression, including second-line salvage therapy and palliative care. The proportion of patients receiving second-line salvage (42%) was based on the BCCA registry in which no patients had data reported on subsequent-line salvage. In the absence of data regarding subsequent treatments received by brentuximab vedotin-treated patients upon progression in SG035-0004, it was assumed that the same percentage of first-line brentuximab vedotin-treated patients received second line salvage as the BCCA patients. In addition, it was assumed that brentuximab vedotin-treated patients who were at a later stage of treatment did not receive further subsequent salvage. All patients were assumed to receive palliative care.

### Sensitivity Analyses

The base case analysis involved a series of modelling assumptions and their impact on the ICER was explored through deterministic sensitivity analyses. These included: selection of different parameter distributions to extrapolate PFS/OS for brentuximab vedotin or conventional chemotherapy, varying the time horizon, assuming no wastage of brentuximab vedotin, use of a different mix of conventional chemotherapy regimens, varying the post-progression costs, varying the cost of SCT, exclusion of costs associated with AE management, and varying utility values for health states. A probabilistic sensitivity analysis was also conducted to address the uncertainty in the parameters used in the model by assigning distributions to input parameters and randomly sampling from these distributions over 1000 Monte Carlo simulations. A cost-effectiveness acceptability curve was used to present the probability that brentuximab vedotin should be considered cost-effective at a wide range of values of willingness-to-pay, since the threshold for willingness-to-pay is not a known value. We do note the probability of cost-effectiveness at a value of 50,000 GBP which is higher than the typically used 20 000 to 30 000 GBP threshold used by NICE, since this situation is one in which there is an extension of life benefit and the intended population is small.[24,25]

The base case analysis included a raw comparison of the SG035-0004 population who had each received one or more prior chemotherapies and had an ECOG performance status of 0 or 1 with patients in the BCCA registry who were receiving standard chemotherapy for first-line salvage and had a range of performance statuses. Two alternative scenario analyses were performed: considering only patients receiving first-line salvage treatment (17 patients receiving brentuximab vedotin and 40 patients receiving conventional chemotherapy) and considering only patients with an ECOG performance status of 0 or 1 (58 patients receiving brentuximab vedotin and 26 receiving conventional chemotherapy). The results of these analyses were compared to the base case to explore potential bias.

## RESULTS

### Clinical Outcomes

Figure 1 shows progression-free and overall survival for brentuximab vedotin and conventional chemotherapy over their respective time periods of observation. At the longest trial follow-up period (55 months) 42% of brentuximab vedotin patients were surviving and 38% in progression-free survival, compared to 25% and 17% of conventional chemotherapy patients respectively. Long-term clinical outcomes from the model are presented in Figure 2. Over the lifetime (30-year) time horizon predicted by the model, brentuximab vedotin showed greater predicted survival over conventional chemotherapy (mean life years, 8.67 vs 3.26 years). The mean time progression free was 5.45 years for patients receiving brentuximab vedotin compared with 2.35 years for patients receiving conventional chemotherapy.

**Figure 1.** Observed OS and PFS for Brentuximab Vedotin and Conventional Chemotherapy

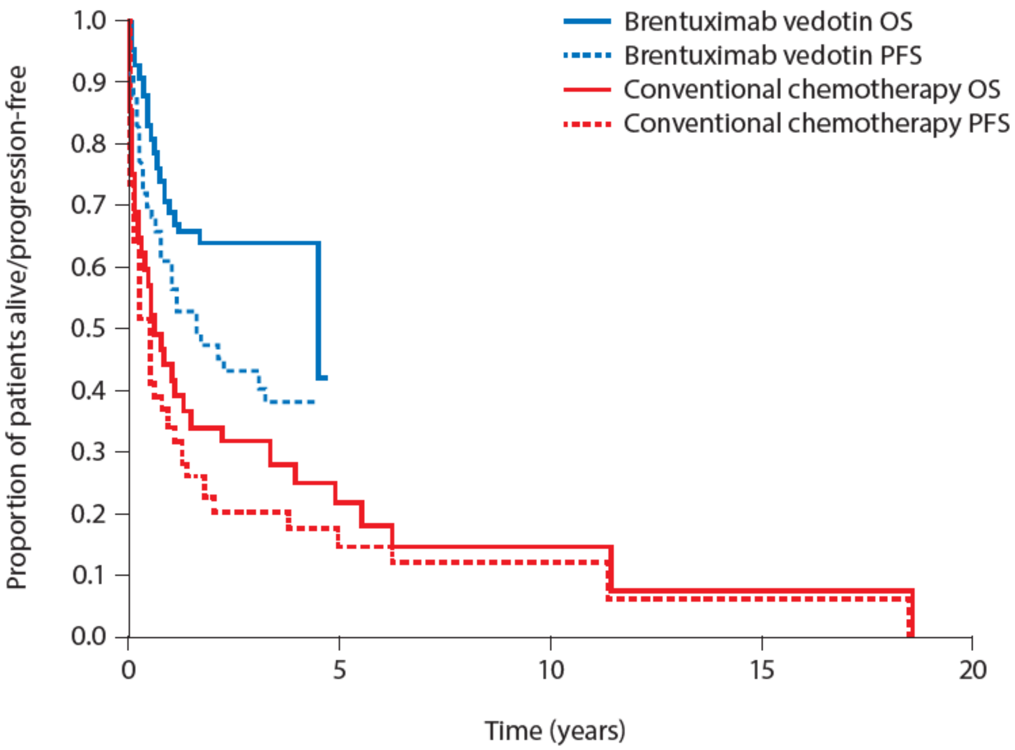

**Figure 2.** Adjusted Predicted OS and PFS for Brentuximab Vedotin and Conventional Chemotherapy

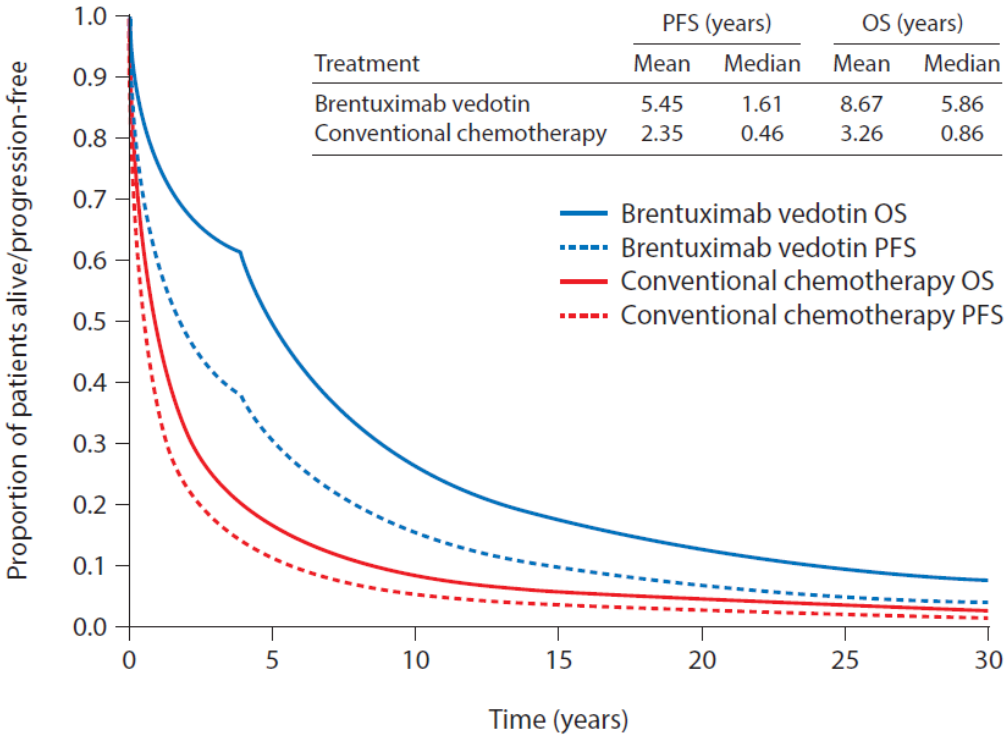

### Cost-effectiveness

The discounted LYs and QALYs by health states, and the discounted costs by resource category for both comparators are presented in Table 2. After discounting, brentuximab vedotin was associated with an OS gain of 3.93 years and an improvement in PFS of 2.31 years. Patients treated with brentuximab vedotin incurred total lifetime costs of £125 258, and the estimated incremental cost of brentuximab vedotin vs conventional chemotherapy was £88 556. The incremental costs of brentuximab vedotin were driven by the higher acquisition cost of brentuximab vedotin (£47 789). Compared with conventional chemotherapy, brentuximab vedotin was estimated to provide a mean gain of 2.50 QALYs, which was mostly accumulated during the pre-progression state (3.50 vs 1.52 QALYs).

Based on the estimated incremental costs and QALYs accrued by brentuximab vedotin relative to conventional chemotherapy, the ICER was approximately £22 500 per LY gained and £35 400 per QALY gained (Table 3).

### Sensitivity Analyses

The cost effectiveness acceptability curves for brentuximab vedotin and conventional chemotherapy, and corresponding cost-effectiveness acceptability frontiers are shown in Figure 3. Probabilistic sensitivity analyses showed that, at a willingness-to-pay threshold of £50 000, the probability that brentuximab vedotin is cost-effective compared with conventional chemotherapy was 86.5%.

In deterministic sensitivity analyses, the ICERs ranged from approximately £28 100 to £61 900 per QALY gained across a range of alternative model assumptions (Table 4). The model was most sensitive to: 1) alternative time horizon; 2) post-progression costs; and 3) the parameter distribution selected to extrapolate PFS for either brentuximab vedotin or conventional chemotherapy.

**Table 2.** Discounted LYs, QALYs and Costs by Resource Category

| | Brentuximab Vedotin | Conventional Chemotherapy |
|---|---|---|
| **Survival** | | |
| Total LYs | 6.52 | 2.59 |
| LYs spent in PFS | 4.19 | 1.88 |
| LYs spent in PPS | 2.33 | 0.71 |
| Total QALYs | 4.25 | 1.74 |
| QALYs spent in PFS | 3.50 | 1.52 |
| QALYs spent in PPS | 0.74 | 0.23 |
| | | |
| **Costs to health care system, total £** | 125 258 | 36 703 |
| Active treatments | 55 340 | 7551 |
| AE management | 108 | 725 |
| SCT | 14 240 | 7028 |
| Off treatment pre-progression | 29 441 | 13 483 |
| Post-progression | 26 128 | 7916 |

AE, adverse event; LY, life year; PFS, progression-free survival; PPS, post-progression survival; QALY, quality-adjusted life year; SCT, stem cell transplant

**Table 3.** Base Case Cost-effectiveness

| Treatment | Costs | LYs | QALYs | ICER (per LY) | ICER (per QALY) |
|---|---|---|---|---|---|
| Brentixumab vedotin | £125 258 | 6.52 | 4.25 | – | – |
| Chemotherapy | £36 703 | 2.59 | 1.74 | – | – |
| Incremental | £88 556 | 3.93 | 2.50 | £22 500 | £35 390 |

ICER, incremental cost-effectiveness ratio; LY, life year; QALY, quality-adjusted life year

Scenario analyses considering only subgroups that were similar on the two key prognostic factors identified opposing effects on the results. The ICER was more favorable for brentuximab vedotin (reduced to approximately £26 800 per QALY gained vs conventional chemotherapy) when only patients given brentuximab vedotin first-line salvage in SG035-0004 were compared to the BCCA registry patients. When only patients with an ECOG performance status of 0 or 1 were considered for both datasets, the ICER for brentuximab vedotin increased to approximately £38 200 per QALY gained.

## DISCUSSION

The cost-effectiveness evaluation presented here was based on the best available evidence for both treatment groups. SG035-0004 provides good quality data to explore the efficacy and cost-effectiveness of brentuximab vedotin.[12-14] However, as a single-arm study, there is no comparator available to allow direct comparisons with standard therapies. In addition, published studies using conventional chemotherapy regimens in the salvage setting do not have adequate data to support development of an economic evaluation model. Therefore, in the absence of randomized comparative data, the final analysis presented a naïve comparison between trial data and ‘real-life’ data.

**Figure 3.** Probability of Cost-effectiveness of Brentuximab Vedotin or Conventional Chemotherapy.
*(a) Cost-effectiveness Acceptability Curves; (b) Cost-effectiveness Acceptability Frontier*

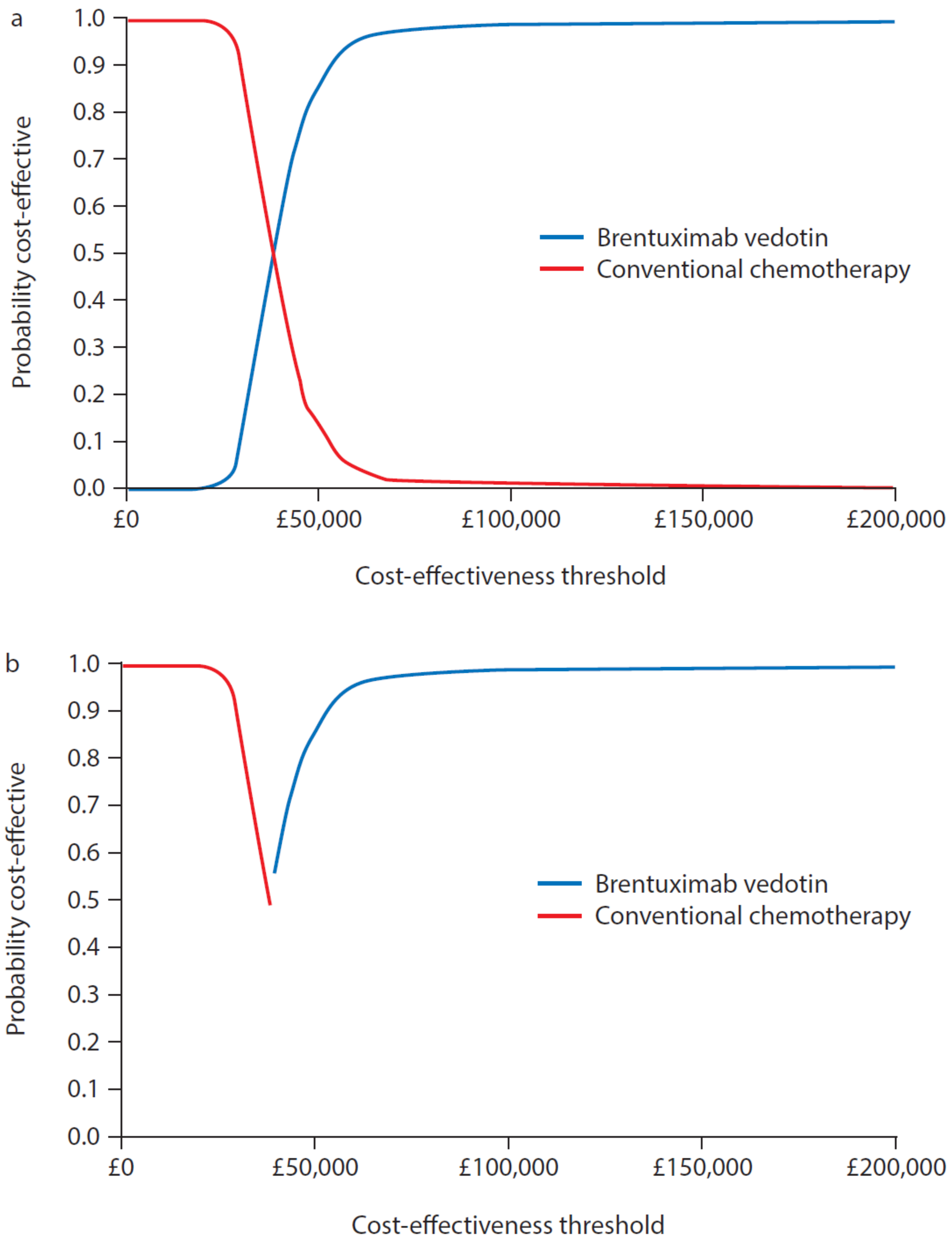

**Table 4.** Deterministic Sensitivity Analyses Results

| Scenario | Base Case | Selection | ICER (per QALY)* | % Change† |
|---|---|---|---|---|
| Base Case | | | £35 400 | |
| Discount rates (costs, benefits) | 0.035, 0.035 | 0.015, 0.015 | £32 400 | –8.39 |
| PFS distribution for brentuximab vedotin | Gamma | Lognormal | £38 400 | 8.41 |
| PFS distribution for brentuximab vedotin | Gamma | Weibull | £37 800 | 6.67 |
| PFS distribution for chemotherapy | Lognormal | Weibull | £41 500 | 17.16 |
| OS distribution for brentuximab vedotin | Gamma | Lognormal | £35 300 | –0.13 |
| OS distribution for brentuximab vedotin | Gamma | Weibull | £35 300 | –0.36 |
| OS distribution for chemotherapy | Lognormal | Gamma | £35 400 | –0.07 |
| Time of receiving SCT since initiating induction chemotherapy | 8.2 months | 6.2 months | £35 400 | 0.05 |
| Time of receiving SCT since initiating induction chemotherapy | 8.2 months | 10.2 months | £35 400 | –0.05 |
| Cost of SCT | base-case value | +25% | £36 000 | 1.76 |
| Cost of SCT | base-case value | +50% | £36 600 | 3.52 |
| Cost of post-progression disease management | £644 per cycle | Doubled | £42 700 | 20.57 |
| Cost of post-progression disease management | £644 per cycle | Zero | £28 100 | –20.57 |
| Basket of conventional chemotherapy | ICE - 33%; ESHAP - 33%; Gem-P - 33% | ICE 30%; ESHAP 14%; CHOP 19%; DHAP 16%; Gem-O 12%; metho 9% | £35 900 | 1.48 |
| Drug wastage | Vial wastage for brentuximab vedotin | No wastage | £32 800 | –7.39 |
| Relative dose intensity | On | Off | £35 390 | 0 |
| Costs of AE management | Included | Excluded | £35 200 | –0.42 |
| Utility difference between pre and post-progression | Base-case value | Utility values for PFS increased by 10% | £32 800 | –7.39 |
| Utility difference between pre and post-progression | Base-case value | Utility values for PFS decreased by 10% | £38 400 | 8.61 |
| Utility decrements for AEs | Included | Excluded | £35 400 | –0.05 |
| Time horizon | 30 years | 5 years | £61 900 | 74.97 |
| Time horizon | 30 years | 10 years | £43 600 | 23.20 |
| Time horizon | 30 years | 20 years | £37 100 | 4.81 |
| Subgroup: first line salvage | All patients | First line salvage only | £26 800 | –24.37 |
| Subgroup: good ECOG PS only | All patients | ECOG PS 0 or 1 only | £38 200 | 7.90 |

*Model-calculated values have been rounded to 3 significant figures.

†Percentage change in ICER per QALY was calculated from unrounded ICER values.

AE, adverse event; CHOP, cyclophosphamide, doxorubicin, vincristine, prednisone; DHAP, dexamethasone, cytarabine (Ara-C), cisplatin; ECOG PS, Eastern Co-operative Oncology Group performance status; ESHAP, etoposide, methylprednisolone, cytarabine, and cisplatin; Gem-O, gemcitabine, oxaliplatin; Gem-P, gemcitabine, cisplatin, and methylprednisolone; ICE, ifosfamide, carboplatin and etoposide; ICER, incremental cost-effectiveness ratio; metho, methotrexate; OS, overall survival; PFS, progression-free survival; SCT, stem cell transplantation.

Extrapolating the available clinical data to a lifetime (30-year) time horizon, the present analysis suggests that brentuximab vedotin was associated with 3.1 years longer mean duration in the PFS health state and an OS improvement of 5.4 years compared with conventional chemotherapy prior to discounting. Discounted to present value and quality adjusted, brentuximab vedotin was associated with 2.5 QALYs gained at an increased total cost to the UK NHS of £88 556, resulting in an ICER of approximately £35 400.

Although the base case ICER is above the £20 000–30 000 threshold for cost-effectiveness typically used by the UK National Institute for Health and Clinical Excellence (NICE), current NICE HTA methodology recognizes that there will be circumstances in which it may be appropriate to recommend the use of treatments with ICERs in excess of the upper end of the range normally approved. In such cases, the treatment must offer an extension life (normally of at least an additional 3 months), at the end of life (normally <24 months life-expectancy), and be licensed or otherwise indicated for small patient populations.[24] Such extended threshold for end-of-life HTAs has previously been shown to be around £50 000 per QALY.[25] Brentuximab vedotin showed an estimated survival advantage of over 5 years compared with conventional chemotherapy (which itself was associated with a life expectancy of less than 24 months). Furthermore, R/R sALCL comprises a small patient population, which is reflected in the designation of brentuximab vedotin as an orphan drug in this indication.[26] Therefore, brentuximab vedotin may be considered a cost-effective use of health care resources in patients with R/R sALCL, providing a higher threshold is applied during health technical appraisal (HTA) for orphan drugs. The results were robust to a wide range of sensitivity analyses. Probabilistic sensitivity analyses found that at willingness-to-pay thresholds greater than approximately £50 000, the probability that brentuximab vedotin is cost-effective compared to conventional chemotherapy is higher than 86.5%. Deterministic sensitivity analyses found that the ICER per QALY gained was robust to the choice of parametric distributions fit to the Kaplan–Meier curves for both OS and PFS, as well as a number of different assumptions about clinical practice and associated costs.

Conventional chemotherapy is considered to include a wide variety of treatment regimens. In our model, the effectiveness of conventional chemotherapy was based on real-world registry data that reported a long historical period of treatment, resulting in the inclusion of a highly heterogeneous group of chemotherapy regimens. Despite this, the majority of treatments used were multi-agent regimens and included platinum-containing, etoposide-containing and gemcitabine-containing regimens; therefore, these are likely to be similar to chemotherapy regimens currently used in clinical practice.[5,11,17] The base case analysis included costs for chemotherapy regimens that are considered current standard in the UK. However, when cost for a wider set of regimens observed as prior salvage for patients entering the brentuximab vedotin trial was considered, the cost-effectiveness of brentuximab vedotin vs conventional chemotherapy remained very similar.

Patients who respond well to treatment and have successful subsequent SCT are expected to experience improved outcomes compared with patients who experience disease progression.[27-29] A slightly higher proportion of patients (29%) in SG035-0004 received subsequent SCT than in the BCCA registry (25%), consistent with a better clinical response with brenuximab vedotin. Our evaluation included the costs and utility impacts of SCT, and survival outcomes were assumed to be represented in the observed data for both groups, with the effect of brentuximab vedotin in allowing more patients to be eligible for SCT included in the evaluation. Moreover, the estimated cost-effectiveness was robust to assumptions about the timing and costs of SCT.

Comparison of clinical trial data and real-world data has some related challenges. Since sALCL is a rare cancer[1], both data sources had small numbers which limited the ability to adjust statistically for sources of potential bias. Eligibility criteria for clinical trials can result in a selected patient sample reflecting the trial efficacy objective(s), rather than demonstrating the effectiveness of treatment in the real-world observational setting. Individual patient populations may differ with regard to important risk factors, both known and unknown, which may introduce bias in the absence of a well-designed direct randomized comparison. In the present study, the two comparator populations differed with regard to two key prognostic factors (presence of prior salvage therapy and ECOG performance status). Our analysis attempted to adjust for the potential biases involved in comparison of these datasets. Because, due to insufficient sample size, the analysis was unable to control for both of these factors simultaneously, the final analysis presented the raw comparison of patients in SG035-0004 and those in the Canadian registry. Line of therapy was found to have the strongest effect, with a bias against brentuximab vedotin. Conversely, having an ECOG performance status of 0 or 1 produced a bias in favor of brentuximab vedotin vs conventional chemotherapy.

The longest trial follow-up period was around 55 months, and at that point there was a large survival advantage considering both overall survival (42% for brentuximab vedotin vs. 25% for conventional chemotherapy) and progression free survival (38% vs. 17%). Survival outcomes were extrapolated beyond the observed outcomes for both treatments using fitted parametric curves. Since there was a high level of censoring in the SG035-0004 data, the survival data curves show a plateau at the end of the observed period[12-14], which made it difficult to estimate survival outcomes beyond the trial period. Extrapolation beyond the SG35-0004 follow-up period assumed the rate of change for brentuximab vedotin to be equal to that in the conventional chemotherapy group, assuming that clinical benefit of brentuximab vedotin does not continue beyond the trial period, which is conservative and consistent with including cost of brentuximab vedotin within the trial period. Notably, when the time horizon was limited to 5 years, the ICER for brentuximab vedotin was increased to approximately £61 900. Extrapolation to 20 years with the rate of change for brentuximab vedotin after the trial period set equal to that for the conventional therapy accrues most of the cost-effectiveness of the full lifetime horizon.

## CONCLUSIONS

The efficacy and safety of brentuximab vedotin in patients with R/R sALCL has already been demonstrated in a phase-2 clinical trial. The present analysis allows consideration of the cost-effectiveness of brentuximab vedotin vs conventional chemotherapy regimens used in routine clinical practice. Brentuximab vedotin has previously been shown to deliver high objective response rates, with a manageable toxicity profile in patients with R/R sALCL.[12-14] Moreover, the cost of brentuximab vedotin should be considered over the full survival period. In the pivotal phase-2 trial, median OS was not reached after a maximum follow up of around 55 months, indicating that a proportion of patients were still receiving benefit for an extended period following a limited duration of treatment.[12-14] Thus, while accommodating uncertainty in our analysis, brentuximab vedotin may provide a valuable treatment choice for this patient population with limited therapeutic options, over the full survival period.

## ACKNOWLEDGEMENTS

The authors would like to acknowledge the writing assistance of Fiona Scott of FireKite, an Ashfield company, part of UDG Healthcare plc, during the development of this manuscript, which was funded by Millennium Pharmaceuticals, Inc.

## CONFLICT OF INTEREST DECLARATION

Margaret Hux: Former employment (Icon Plc); consultant/advisory role and research funding (Millennium Pharmaceuticals Inc., Cambridge, MA, USA, a wholly owned subsidiary of Takeda Pharmaceutical Company Limited)

Denise Zou: Employment (Icon Plc); consultant/advisory role and research funding (Millennium Pharmaceuticals Inc., Cambridge, MA, USA, a wholly owned subsidiary of Takeda Pharmaceutical Company Limited)

Esprit Ma: Former employment (Millennium Pharmaceuticals Inc., Cambridge, MA, USA, a wholly owned subsidiary of Takeda Pharmaceutical Company Limited); stock ownership (Takeda Pharmaceutical Company Limited)

Peter Sajosi: Employment (Millennium Pharmaceuticals Inc., Cambridge, MA, USA, a wholly owned subsidiary of Takeda Pharmaceutical Company Limited)

Andreas Engstrom: Employment (Millennium Pharmaceuticals Inc., Cambridge, MA, USA, a wholly owned subsidiary of Takeda Pharmaceutical Company Limited)

Ross Selby: Employment (Millennium Pharmaceuticals Inc., Cambridge, MA, USA, a wholly owned subsidiary of Takeda Pharmaceutical Company Limited)

Eugene Benson: Employment (Millennium Pharmaceuticals Inc., Cambridge, MA, USA, a wholly owned subsidiary of Takeda Pharmaceutical Company Limited)

Andrew Briggs: Employment (Icon Plc); honoraria and consultant/advisory role (Bristol-Myers Squibb, GlaxoSmithKline, Sanofi, Millennium Pharmaceuticals Inc., Cambridge, MA, USA, a wholly owned subsidiary of Takeda Pharmaceutical Company Limited)

Vijayveer Bonthapally: Employment (Millennium Pharmaceuticals Inc., Cambridge, MA, USA, a wholly owned subsidiary of Takeda Pharmaceutical Company Limited); stock ownership (Takeda Pharmaceutical Company Limited)
